# Normalizing a large number of quantitative traits using empirical normal quantile transformation

**DOI:** 10.1186/1753-6561-1-s1-s156

**Published:** 2007-12-18

**Authors:** Bo Peng, Robert K Yu, Kevin L DeHoff, Christopher I Amos

**Affiliations:** 1 Department of Epidemiology, The University of Texas, M.D. Anderson Cancer Center, 1155 Pressler Boulevard, Unit 1340, Houston, Texas 77030, USA

## Abstract

Variance-components and regression-based methods are frequently used to map quantitative trait loci. The normality of the trait values is usually assumed and violation of this assumption can have a detrimental effect on the power and type I error of such analyses. Various transformations can be used, but appropriate transformations usually require careful analysis of individual traits, which is not feasible for data sets with a large number of traits like those in Problem 1 of Genetic Analysis Workshop 15 (GAW15). A semiparametric variance-components method can estimate the transformation along with the model parameters, but existing methods are computationally intensive. In this paper, we propose the use of empirical normal quantile transformation to normalize the scaled rank of trait values using an inverse normal transformation. Despite its simplicity and potential loss of information, this transformation is shown, by extensive simulations, to have good control of power and type I error, even when compared with the semiparametric method. To investigate the impact of such a transformation on real data sets, we apply variance-components and variance-regression methods to the expression data of GAW15 and compare the results before and after transformation.

## Background

The rapid expansion of the size of data sets poses new challenges to mapping genes associated with quantitative traits. Facing massive amounts of data, it is no longer feasible to analyze individual traits or genotypes manually. Many methods, though theoretically advantageous, cannot be used due to their requirements of user intervention or a high demand for computing power. Automatic and efficient algorithms become more and more important. In this paper, we seek such an algorithm for the normalization of a large number of quantitative traits.

Many models are used to map genes responsible for quantitative traits. Some of the most commonly used ones are Haseman-Elston regression, variance components [1], and variance regression [2]. All these methods perform optimally when the trait values of family members follow a multivariate normal distribution. Violation of this assumption can have detrimental effects on the type I error and power, particularly for variance-components methods [3]. Various methods have been proposed to transform trait values, including simple transformations such as square root and logarithm transformations, and more advanced ones such as Box-Cox transformation and rank-based transformations. However, the choice of transformation is often arbitrary, and different choices can lead to conflicting results. Diao and Lin [4] proposed a method that treats the transformation as part of the parameter space and estimates the transformation along with other parameters. The resulting transformation is rank based and is asymptotically efficient among all order-preserving transformations. However, existing implementations are computationally intensive.

None of these methods can be used for data sets with a large number of quantitative traits, as those in Problem 1 of Genetic Analysis Workshop 15 (GAW15). In this data set, there are 3554 traits and 2882 SNP markers across 23 chromosomes, collected for 194 individuals in 14 families. Among all 3554 traits, almost half (45.8%) of them fail the Anderson-Darling normality tests at the 0.05 significance level. Given the large number of traits, it is not possible to analyze each trait and transform them according to their distributions or to use the computationally intensive semiparametric algorithm. Tossing away half of the traits because the uncertain impact of non-normality on subsequent data analyses is not a good idea either, even when there is an abundance of them.

We propose the use of a rank-based transformation method called the empirical normal quantile transformation (ENQT). This method ranks the trait values and scales the ranks to (-1, 1). It then transforms the scaled ranks to a normal distribution using an inverse normal transformation. This method is computationally efficient and can be blindly applied to all the quantitative traits, resulting in perfectly normal trait values provided there are few tied values. The major concern is that ENQT uses only the rank information of the original trait values so it may not perform well compared to customized transformations for each trait, or transformation obtained from the semiparametric quantitative trait locus (SQTL) method [4].

In this paper, we test the impact of ENQT on the power and type I error of the variance component method using extensive simulations. Then, we apply ENQT to the GAW15 data set and study the impact of the transformation on the subsequent data analyses.

## Methods

### Simulations to test the impact of ENQT on power and type I error

The parental trait is determined by *H*(*Y*_*ij*_) where

*Y*_*ij *_= *β*_1_*X*_1*ij *_+ *β*_2_*X*_2*ij *_+ *g*_*ij *_+ *G*_*ij *_+ *e*_*ij*_

is the original trait value of individual *j *in family *i*. *H*(*Y*) = *e*^1+*y *^+ (5 + *y*)^2 ^transforms *Y*_*ij *_to a distribution with an average kurtosis of 54.1 and skewness of 4.98 if *Y*_*ij *_is normal *N*(0, 1.5). *X*_1*ij *_and *X*_2*ij *_are fixed covariates mimicking standardized age (*N*(0, 1)) and sex (male or female with equal probability) with *β*_1 _= -0.5 and *β*_1 _= 0.5. *g*_*ij *_is the major gene effect determined by the true QTL, which assumes value *-a*, 0, or *a *for genotype *AA*, *Aa*, or *aa*, respectively. The major genetic variance is therefore *σ*_*g*_^2 ^= 2*pqa*^2 ^= $\frac{a^{2}}{2}$. *G*_*ij *_is the polygenic effect that follows a normal distribution with mean 0 and variance *σ*_*G*_^2^. *e*_*ij *_is a normal random environmental effect with mean of 0 and variance of *σ*_*e*_^2^. The genetic heritability *h*^2 ^and major gene heritability *h*_*g*_^2 ^are calculated as *h*^2 ^= (*σ*_*g*_^2 ^+ *σ*_*G*_^2^)/*σ*^2 ^and *h*_*g*_^2 ^= *σ*_*g*_^2^/*σ*^2^, respectively, where *σ*^2 ^= *σ*_*g*_^2 ^+ *σ*_*G*_^2 ^+ *σ*_*e*_^2 ^is the total sample variance. The trait of offspring is determined in a similar way but the offspring's polygenic effects are determined by $\frac{G_{ij}^{P}+G_{ij}^{M}}{2}+N\left(0,\frac{\sigma_{G}^{2}}{2}\right)$, where *G*_*ij*_^*P *^and *G*_*ij*_^*M *^are the paternal and maternal polygenic effects of the parents, respectively.

We simulated the same six schemes as those in Diao and Lin [4]. Namely, we set *σ*_*g*_^2^, *σ*_*G*_^2^, and *σ*_*e*_^2 ^to (0, 1, 1), (0.2, 0.8, 1), (0.4, 0.6, 1), (0, 0.6, 1.4), (0.2, 0.4, 1.4), and (0.4, 0.2, 1.4) for schemes *a *through *f*, respectively. Among these schemes, schemes *a *and *d *serve as null hypotheses because their major gene heritabilities are 0. For each setting, we generated 20,000 data sets. The variance-components method was applied to original (*H*(*Y*_*ij*_)), perfectly back-transformed (*Y*_*ij*_), and ENQT-transformed trait values. The SQTL method was also applied to the original trait values. The percentage of simulations with *p*-values less than 5%, 1%, and 0.1% are reported.

### Application to Problem 1 of GAW15

We took the expression data of Problem 1 of GAW15 and transformed each trait by ENQT. The resulting traits are normal with high *p*-values (>0.99) in normality tests. Besides descriptive statistics (mean, variance, skewness, and kurtosis), we applied the Anderson-Darling normality test and variance-components method to estimate polygenic heritability. Using these initial statistics, we chose several groups of traits that are:

1. Normally distributed (*p*-value of Anderson-Darling normality test >0.7) with before-transformation heritability >0.3. This group has 81 traits.

2. Significantly non-normally distributed with *p*-value of Anderson-Darling normality test <0.0001 and with before-transformation heritability >0.4. This group has 43 traits.

3. Having high heritability (>0.6) before transformation. This group has 37 traits.

4. Having a high difference in heritability before and after transformation (>0.1). This group has 49 traits.

5. Having low difference of heritability (<0.001), with before-transformation heritability >0.3. This group has 49 traits.

We use heritability as a criterion because traits with low heritability may not be of interest. These groups sometimes overlap. For example, there are 16 common traits in the non-normal and high heritability groups, indicating potential exaggeration of the estimates of heritability due to non-normality.

For traits in these groups, we performed and compared full genome-wide scanning using variance component [1] and variance regression [2] methods, and compared the LOD scores at the SNP markers before and after transformation.

## Results

### Impact of ENQT transformation on power and type I error

Table 1 lists the percentages of simulations with *p*-values less than the given significance levels. The four columns correspond to trait values after a perfect back-transformation, no transformation, and ENQT transformation, all analyzed by variance components method; and analyzed by SQTL. Only results for simulations with two offspring per family are reported.

**Table 1:** Power and type I error of simulations with varying level of heritability for sib pairs

	Perfect transformation			No transformation			ENQT transformed			Semiparametric QTL		
				
Model	5%	1%	0.10%	5%	1%	0.10%	5%	1%	0.10%	5%	1%	0.10%
*a*^a^	4.96	1.02	0.08	11.1	3.63	0.93	4.89	1.05	0.09	2.42	0.98	0.25
*b*	13.97	3.71	0.51	15.9	5.58	1.62	14.06	3.81	0.51	8.85	3.87	1.32
*c*	31.69	11.98	2.45	22.92	7.95	1.83	31.55	11.96	2.45	23.72	12.67	5.52
*d*^a^	4.69	0.48	0.01	4.68	1.14	0.3	4.71	0.5	0.02	1.94	0.45	0.09
*e*	11.95	1.96	0.06	7.05	1.62	0.39	11.9	1.94	0.06	6.26	1.91	0.3
*f*	24.54	6.02	0.39	10.2	2.56	0.67	24.58	5.88	0.38	15.75	5.95	1.67

^a^These replicates reflect the null model for which there is no major gene effect.

Scheme *a *and *d *reflect the null model for which there is no major gene effect. Non-normality causes highly inflated type I error for scheme *a *when no transformation is applied, but not for scheme *d*. This is because departure from normality only causes excess false positives when there is residual correlation in the relatives not explained by the major locus and kurtosis (or perhaps skewness), which is the case for *a *but not *d *[5]. The variance-components method seems to have a lower-than-nominal level for simulation of sib pairs and a higher-than-nominal level for larger sibships (results not shown). In either case, ENQT provides the correct type I error level. The result of SQTL is ambiguous because it shows lower-than-nominal level type I error at 0.05 level but higher at 0.001 level. For other schemes, it is clear that the power of the variance-components method is greatly affected by non-normality. The variance-components method using ENQT transformation has consistently better power than the SQTL method. As a matter of fact, ENQT transformation achieves roughly the same power as the perfect back-transformation in all cases while preserving the type I error rate.

### GAW data set

ENQT transformation can have significant impacts on the analyses of quantitative traits. Using trait 209785_s_at as an example, we compare the LOD scores at the SNP markers on all autosomes, before and after ENQT transformation. This trait has kurtosis of 1.54 and skewness of -1.17. Its heritability measures 0.41 before transformation and 0.44 afterward. After ENQT transformation, two large peaks on chromosome 9 and 11 decrease dramatically, with maximum decreases of LOD scores from 3.43 to 1.61 and from 2.63 to 1.34, respectively. Smaller but sometimes wider peak changes can also be found on chromosome 1 (from 2.03 to 0.66), 5 (from 1.74 to 0.10), and 8 (from 1.50 to 0.14). On the other hand, the transformation magnifies a narrow peak on chromosome 11 (from 2.06 to 3.63) and induces a wide peak on chromosome 19 (from 1.15 to 3.35).

Table 2 summarizes the change of LOD scores of the genome-wide scan before and after ENQT transformation. For each group, we calculate mean difference of LOD scores, and mean number of SNP markers that have become significant (with LOD > 1, 2, or 3) after transformation and the mean number of SNP markers that are no longer significant (with LOD < 1, 2, or 3). For example, for traits that are significantly non-normal, if we use LOD = 3 as the cut-off value, on average 16.8 markers are no longer significant after transformation and 8.1 markers become significant. Consecutive markers that form wide peaks are counted individually.

**Table 2:** Change of LOD scores before and after ENQT transformation averaged over all traits in the groups

		Average no. SNPs with LOD score above/below:					
		
Method^a^	LOD difference^b^	above 1	below 1	above 2	below 2	above 3	below 3
Normal traits (81)							
vc	0.020	6.3	10.1	4.0	4.6	4.6	2.7
reg	0.022	6.9	9.1	4.6	3.7	2.8	2.6
Non-normal traits (53)							
vc	0.107	22.6	71.4	9.2	31.4	8.1	16.8
reg	0.086	31.4	36.4	8.9	13.6	6.1	8.3
High difference in heritability (49)							
vc	0.105	25.7	77.9	11.2	35.5	16.5	20.9
reg	0.085	35.7	30.4	9.9	15.6	6.0	7.9
Low difference in heritability (49)							
vc	0.043	14.0	22.0	5.5	12.3	6.5	5.0
reg	0.038	12.5	15.3	6.6	5.8	6.5	8.0
High heritability (37)							
vc	0.110	28.7	86.1	13.7	47.0	8.3	19.3
rev	0.060	21.9	21.3	8.2	14.2	5.4	6.7

^a^*vc *and *reg *stand for variance-components and variance-regression methods, respectively.

^b^Difference in LOD scores averaged over all markers

ENQT transformation has a different impact on traits in different groups. The average difference of LOD scores, and the number of changed markers of the variance-components method are larger than those of the variance-regression method. This suggests that the variance-components method is more sensitive to non-normality than the variance-regression method.

For both mapping methods, ENQT transformation causes more reduced LOD scores than increased LOD scores, which may contribute to decreased false-positive rates. Among these five groups, the normal group has the least LOD score changes followed by the group with low changes in heritability. Groups with high heritability differences, significantly non-normal and high heritability, have large changes in LOD scores. Note that these three groups overlap and have seven traits in common. These traits are 201481_s_at, 203032_s_at, 204428_s_at, 205048_s_at, 209480_at, 219843_at, and 65588_at.

## Discussion

In this paper, we show that normalization has a significant impact on the QTL mapping, using variance-components and regression-based methods. We also show that ENQT transformation is an efficient transformation that outperforms traditional and semiparametric transformation methods. This method is especially suitable for problems with a large number of traits for which customizing the transformation for each trait becomes infeasible.

Our simulations show that ENQT transformation performs similarly to a perfect back-transformation and outperforms the SQTL method, which has been proven to have better power than square-root and logarithm transformations for this particular example [4]. However, this may reflect the particular simulation method and parameters we use. SQTL is rank based, is proven to be asymptotically efficient among all transformations that keep the order of the original trait values, and has a power similar to the traditional variance-components method with normally distributed data. These facts, along with the facts that ENQT is also rank based and produces normally distributed trait values, indicate that ENQT should yield a similar profile when compared with SQTL. The poor performance of SQTL compared with ENQT could reflect difficulties in maximization over a higher-dimensional likelihood space.

It should be pointed out that the optimal transformation does not have to normalize the trait values. In the cases when there are strong and discrete covariate effects, *Y*_*ij *_may be bi-normal or some other non-normal distribution. SQTL may perform better in such cases because it assumes conditional normality and can in theory normalize trait values after removing covariate effects.

GAW15 Problem 1 has fewer and larger families than what we have simulated, and our simple transformation may discard delicate within-family structures. For example, we have seen traits that are associated with age, resulting in differences in normality test results for each generation as compared to the entire population. However, given the small sample size, it seems impractical to perform normalization at a finer scale.

The results presented use Anderson-Darling normality test, even though other normality tests may produce different results. We repeated the normality tests using Sharpiro-Wilk's test, which is suitable for samples of size less than 200. The two tests largely agree with each other, and there are only a few changes to the five groups of markers we chose.

## Conclusion

In summary, we show that normalization can have a strong impact on the results of variance-components and regression-based method and ENQT can be a good candidate to blindly transform a large number of quantitative traits. It is therefore recommended that results based on untransformed data be repeated with normalized trait values using ENQT method. If there are significant differences, caution should be taken when making statistical inferences.

## Competing interests

The author(s) declare that they have no competing interests.
